# Examining Portuguese High School Students’ Attitudes Toward Physical Education

**DOI:** 10.3389/fpsyg.2020.604556

**Published:** 2020-11-26

**Authors:** Paulo Pereira, Fernando Santos, Daniel A. Marinho

**Affiliations:** ^1^School of Higher Education, Polytechnic Institute of Porto, Porto, Portugal; ^2^School of Higher Education, Polytechnic Institute of Viana Do Castelo, Viana Do Castelo, Portugal; ^3^Department of Sport Sciences, University of Beira Interior, Covilhã, Portugal; ^4^Research Centre in Sports, Health and Human Development, CIDESD, Covilhã, Portugal

**Keywords:** school, pedagogy, curriculum, extracurricular sports, physical activity

## Abstract

Portugal ranks fourth among countries with the highest rate of overweight population, considering that 67.6% of the Portuguese population over the age of 15 is overweight or obese. To our knowledge, limited studies have investigated students’ attitudes toward physical education in Portugal. Such research is necessary because it can provide valuable insights for policy and application in the curriculum development for physical education, which may eventually increase participation in physical and sports activities. This study analyzed students’ attitudes toward physical education (PE) according to sociodemographic variables, including grade level, socioeconomic status, and gender, and their participation in extracurricular sports activities and respective PE grades. The sample comprised 476 high school students (from the 7th, 8th, and 9th-grade levels) from five public schools located in Portugal. The Students’ Attitudes toward Physical Education Questionnaire was adapted and validated for use with Portuguese students as a two-factor model. Findings show that students generally have a moderately positive attitude toward PE. However, students’ positive attitudes tend to decrease throughout high school, which is particularly significant in the 9th grade. Furthermore, students’ attitudes are influenced by gender, extracurricular sports practice, and grades. These findings may help stakeholders reflect on how to frame PE in a more meaningful way to create a solid foundation for maintaining an active lifestyle throughout life. Implications for further research and practice are discussed.

## Introduction

Previous research has shown an increase in sedentary behavior among young people over the last 20 years (Li et al., 2014), which has contributed to the rise in global obesity rates. The Organization for Economic Cooperation and Development (OECD, 2019) has reported that obesity has been gradually increasing since the early 2000s among adults; specifically, 67.6% of the Portuguese population over the age of 15 is considered overweight or obese. Portugal, together with Finland, ranked fourth in the OECD countries with the highest rates of overweight population, preceded by Chile (74.2%), Mexico (72.5%), and the United States (71%) who are in the top three.

The decrease in participation of young people in physical activities and sports, which has a significant impact on the overall health (Guthold et al., 2020), stresses the need to identify the factors that contribute to increasing the level of participation. Therefore, compulsory physical education (PE) in Portugal may be essential. A primary goal of PE is to encourage young people to adopt active lifestyles by teaching them to engage in persistent and meaningful lifelong participation in physical activities and sports (Marttinen et al., 2018). This means that PE should provide youth with substantial physical activity in the school environment since it is particularly important for those who engage in low levels of daily physical activity (Hu et al., 2014). Additionally, PE also creates firm foundations for participation in sports activities outside school (McKenzie, 2003; Wallhead and Buckworth, 2004; Constantinides and Silverman, 2018). However, considering this objective, we might find PE classes to be insufficient to meet the recommendations of the World Health Organization (2010) and the American College of Sports Medicine (2018) to promote substantial autonomous extracurricular sports activities (Sierra-Díaz et al., 2019). For instance, in Portugal, students from 5th to 12th year (i.e., students aged above 11 years) have 150 min per week of compulsory PE, which does not meet the standards set by the World Health Organization (2010).

Previous studies have highlighted the influence of some variables on students’ subsequent participation in physical activities and sports outside the school context (Subramaniam and Silverman, 2000; Jaakkola et al., 2017). One of these variables is *attitudes* (Mercier et al., 2017). Eagly and Chaiken (1993, p. 1), in their seminal work, state that attitudes can be understood as a hypothetical construct referring to the “psychological trend that is expressed in a favorable or unfavorable assessment of a specific entity.” A long-term goal of PE programs worldwide is to develop positive attitudes toward PE (Donovan et al., 2015). Thus, several studies have shown that the development of positive attitudes toward PE can help young people engage in physical activities outside school and promote active lifestyles during their school years (Hagger et al., 2003; Phillips and Silverman, 2015; Solmon, 2015) and adulthood (Telama et al., 2005; Subramaniam and Silverman, 2007).

Several models facilitate our understanding of how students’ attitudes influence behavior, such as the theory of reflected action/theory of reasoned action (TRA; Fishbein and Ajzen, 1975), the theory of planned action/theory of planned behavior (TPB; Ajzen, 1985), and the reasoned action model (RAM; Fishbein and Ajzen, 2010). Most studies on students’ attitudes toward PE developed in the 21st century were guided by TRA (Silverman, 2017), which supports the notion that all behavior is a choice among several alternatives, and therefore, behavioral intention (i.e., predisposition toward a behavior) is the best predictor of behavior. Behavioral intention is determined by two important factors: an individual’s attitude toward the behavior (i.e., personal influence on behavior) and subjective norms (i.e., social pressures that affect behavior).

In recent decades, the number of studies that focus on students’ attitudes toward PE (Silverman and Subramaniam, 1999; Subramaniam and Silverman, 2000; Chung and Phillips, 2002; Koca and Demirhan, 2004; Li et al., 2014) has increased after the review article published by Silverman and Subramaniam (1999) suggested the need to further develop the field and strive for quality PE. Additionally, they revealed that most previous studies used non-validated instruments, qualitative methodologies, and were conceptually fragile (Silverman and Subramaniam, 1999). Recently, Silverman (2017) reinforced the need to develop theory-based research that involved more rigorous designs. As such, the construction and validation of the Students’ Attitudes toward Physical Education Questionnaire (SAtPE; Subramaniam and Silverman, 2000) motivated many studies on students’ attitudes toward PE.

The SAtPE (Subramaniam and Silverman, 2000) was designed to assess students’ attitudes toward PE, and has favorable psychometric properties of validity and fidelity (Subramaniam and Silverman, 2000; Montalvo and Silverman, 2008; Donovan et al., 2015; Constantinides and Silverman, 2018). Over the past 20 years, most studies that have used SAtPE have found that students have moderate to high positive attitudes toward PE. However, it should be noted that most of these studies were conducted in the United States (Subramaniam and Silverman, 2000, 2007; Montalvo and Silverman, 2008; Zeng et al., 2011; Donovan et al., 2015; Phillips and Silverman, 2015; Scrabis-Fletcher et al., 2016; Mercier et al., 2017; Scrabis-Fletcher and Silverman, 2017; Marttinen et al., 2018), while only a few have been conducted in European (Lazarević et al., 2015; Constantinides and Silverman, 2018; Evangelou and Digelidis, 2018; Orlić et al., 2018) and Asian countries (Koca and Demirhan, 2004; Hu et al., 2014). Moreover, research in non-English-speaking countries, which include diverse education systems, is still scarce. Considering the increase in the number of obese individuals and those who do not have an active lifestyle across European countries (OECD, 2019), more insight about students’ attitudes toward PE is necessary to provide important implications for policy, research, and practice.

According to the literature, several variables determine students’ attitudes toward PE, such as grade level, gender, and family’s socioeconomic status. Regarding the educational level, most studies suggest that students’ positive attitudes toward PE decrease after high school (Subramaniam and Silverman, 2000, 2007; Hu et al., 2014; Lazarević et al., 2015; Scrabis-Fletcher et al., 2016; Mercier et al., 2017; Evangelou and Digelidis, 2018; Marttinen et al., 2018). Concerning gender, most studies did not show statistically significant differences between males and females (Subramaniam and Silverman, 2000, 2007; Hu et al., 2014; Scrabis-Fletcher et al., 2016; Marttinen et al., 2018; Orlić et al., 2018), while others have reported that male students have more positive attitudes than female students (Lazarević et al., 2015; Mercier et al., 2017). Finally, a study conducted at a North American high school (Zeng et al., 2011) found no significant differences in attitudes toward PE based on students’ socioeconomic status. Nevertheless, research on the impact of socioeconomic status on students’ attitudes toward PE is still scarce.

Recently, interest in the association between students’ attitudes toward PE and their participation in extracurricular physical activities has been increasing. The development of positive attitudes toward PE has been considered a determinant for young people to remain active outside the school environment (Hagger et al., 2003; Solmon, 2003; Phillips and Silverman, 2015), in high school (Lazarević et al., 2015; Orlić et al., 2018), or secondary school (Koca and Demirhan, 2004).

Attitudes toward PE are also influenced by contextual factors (Phillips and Silverman, 2015), such as teachers and the curriculum (Subramaniam and Silverman, 2000; Silverman, 2011; Mercier et al., 2017). Silverman (2011) seminal work highlighted curriculum as the most influential factor in developing students’ attitudes toward PE. Additionally, Luke and Sinclair (1991) alluded to the fact that teachers’ behaviors constitute the second most decisive factor in students’ negative attitudes toward PE.

The present study was conducted in Portugal. Similar to other European countries, compulsory education in Portugal last for 12 years. PE is part of the curriculum in the first 4 years (first to fourth-grade levels; children aged between 6 and 10 years). However, in some cases, it is disregarded by generalist teachers, and engaging in various extracurricular physical activities and sports is not compulsory. Conversely, PE is a compulsory class for 5th to 12th grade. Students’ grades in PE are also considered in their weighted average and application to universities.

To our knowledge, studies that investigate students’ attitudes toward PE in the Portuguese context are scarce, and there is a lack of validated instruments that focus on this field. Therefore, this study proposed to (a) validate the SAtPE (Subramaniam and Silverman, 2000) for use in the Portuguese population, (b) analyze Portuguese high school students’ attitudes toward PE, and (c) examine the influence of sociodemographic variables (i.e., educational and socioeconomic level and gender), extracurricular sports participation, and school performance in PE (i.e., grade attained) on students’ attitudes toward PE. The following hypothesis guided the present study: (1) high school students’ have a moderately positive attitude toward PE; (2) high school students’ attitudes decrease throughout grades in PE; (3) male high school students have more positive attitudes toward PE than their female counterparts; (4) high school students with higher socioeconomic status have more positive attitudes toward PE than students with lower socioeconomic status; (5) high school students who engage in extracurricular sports activities have higher positive attitudes toward PE than those who do not; (6) high school students with higher grades have more positive attitudes toward PE.

## Materials and Methods

### Study Design

Based on Ato et al.’s (2013) classification system for research designs in psychology, our study design represents empirical research purposes and involved a retrospective design or *ex post facto*. Thus, we examined causal relationships between independent and dependent variables. A correlational design was used since the independent and dependent variables were not manipulated. Furthermore, sampling was not determined based on these variables (Tuckman, 2002).

### Study Phases

The present study involved two phases. In the first phase, we validated the SAtPE by conducting several analytical steps, as suggested by previous research (Subramaniam and Silverman, 2000). This process involved the translation of the instrument, the use of confirmatory factor analysis, and an assessment of the questionnaire’s fidelity. In the second phase, after the validation process, the questionnaire was administered to a large sample of high school students.

### Sample

A non-probabilistic sampling method was utilized to recruit participants. A convenience sample of 476 students was included in the present study. The participants were students from the 7th, 8th, and 9th grades who were recruited from five urban high schools in five districts in northern and central Portugal. Of the sample, 52.3% (*n* = 249) were male and 47.7% (*n* = 227) were female. The students’ ages ranged between 12 and 17 years (*M* = 13.38; SD = 0.95). Regarding the socioeconomic status, 47.7, 38.4, and 13.9% came from middle, low, and high-class families, respectively. Socioeconomic status was determined according to the criteria proposed by Simões (2000) for the Portuguese population. Moreover, 65.5 and 58.1% of male and female students, respectively, were involved in extracurricular physical activities and sports. Furthermore, 38.7, 49.8, and 9.2% had a final grade of 3, 4, and 5, respectively. Only 2.3% failed in PE and had a final grade of 2.

### Procedure

Before conducting the present study, ethical approval was attained from the Ministry of Education (Office of Statistics and Education Planning), and school directors were presented with the study objectives, scope, and implications. PE teachers, parents, tutors, and students were briefed about the study, and informed consent was obtained. Data were collected between March 2019 and June 2019. The SAtPE was administered during the beginning or the end of a PE class, with at least one researcher present to provide any clarifications as needed. The questionnaire included information about the purpose of the study and instructions for the participants. Participants were informed that they were not being evaluated (scored) to avoid the effects of social desirability. Additionally, they were notified about the confidentiality of the questionnaire data, and that PE teachers would not have access to the results.

### The SAtPE Questionnaire

It comprises 20 items, 8 of which are negatively worded. Responses are provided using a five-point Likert scale ranging from 1 (*totally disagree*) to 5 (*totally agree*), with scores ranging from 20 to 100, with higher scores indicating more positive attitudes toward PE. Based on the previous notions, attitudes are based on two key components (cognitive or perceived utility and affective or enjoyment) (Subramaniam and Silverman, 2007), which contain two sub-factors each (teacher and curriculum). The SAtPE (see section “Appendix” for the complete questionnaire) involves 10 items associated with cognitive or perceived utility (items 4, 6, 7, 8, 10, 13, 14, 16, 17, and 18) and 10 items associated with the affective or enjoyment component (items 1, 2, 3, 5, 9, 11, 12, 15, and 19).

### Translation

Permission was granted from the authors who constructed the original questionnaire to validate it in the Portuguese context. We used a back-translation method to ensure the appropriateness of the translation. It is a most widely used method in social sciences to assess the appropriateness and clarity of language (Douglas and Craig, 2007). We requested two university professors who were fluent in English language to translate the questionnaire into Portuguese. These two translations were subsequently compared with no major differences between them. We then asked another university professor to translate the final Portuguese version into English. The original version of the instrument was compared with the back-translated version with no major differences; hence, the questionnaire was considered suitable. Subsequently, a pilot study was conducted with a group of 30 students to test the clarity of language and appropriateness of the items.

This subsample of students was not included in the main study. The average of the students was 13.53 years (SD = 1.13) with 47.7% males and 53.3% females. No issues were raised regarding the items’ appropriateness and clarity of language.

### Data Analysis

In the first phase of the study, we examined SAtPE’s psychometric properties using the *Analysis of Moment Structures* software (AMOS; version 24). First, univariate and multivariate normality were determined by the asymmetry coefficients (Sk; |Sk| < 3), kurtosis (Ku; |Ku| < 10), and Mardia’s (1970) coefficient. Asymmetry values higher than 3 and kurtosis values higher than 10 need transformation (Kline, 2016). Furthermore, Mardia’s coefficient is considered appropriate when the value is lower than *p* (*p* + 2), which represents the number of observed variables (Bollen, 1989). Second, a confirmatory factorial analysis (CFA) was conducted using the maximum likelihood estimation method. This procedure assessed whether the SAtPE followed the factorial structure proposed in the original version (Subramaniam and Silverman, 2000). Third, model adequacy was tested using absolute and relative fit indexes recommended in previous literature (Hu and Bentler, 1999; Jackson et al., 2009; Byrne, 2010; Marôco, 2014). Absolute fit indexes included Chi-square (χ^2^), Chi-square and degrees of freedom ratio (χ^2^*/gl*), and the goodness of fit index (GFI). Conversely, the relative fit indexes were the comparative fit index (CFI), normed fit index (NFI), and Tucker-Lewis index (TLI). The following incremental indexes were also utilized: root mean square error of approximation (RMSEA) and root mean square residual (RMSR). Previous studies have suggested that there is appropriate model adequacy when χ^2^/gl < 2; GFI ≥ 0.95; CFI ≥ 0.95; NFI ≥ 0.95 e TLI ≥ 0.95; RMSEA < 0.06; RMSR < 0.08. However, these values have been considered acceptable: χ^2^*/gl* < 5, GFI > 0.90, CFI > 0.90, NFI > 0.90, TLI > 0.90, RMSEA < 0.10, RMSR < 0.10 (Hu and Bentler, 1999; Byrne, 2010; Kline, 2016). Fourth, individual reliability (λ^2^) and composite reliability (CR) were tested to verify construct reliability. On the other hand, concurrent reliability was tested through the average variance extracted (AVE) of each factor with a cut-off point of 0.50 (Hair et al., 2018). Finally, the discriminant validity of each factor was assessed through a comparison between the AVE and average shared squared variance. We assessed the temporal stability of SAtPE through a test-retest procedure. Thus, this procedure involved 46 students who filled the questionnaire on two occasions with a 2-week interval. Results were analyzed using Pearson’s correlation coefficient. According to Keszei et al. (2010), a 10–14 days interval between the test and retest is deemed appropriate. Furthermore, they also consider 0.70 and 0.80 as acceptable reliability coefficients.

In the second phase of the study, data were subjected to several procedures using the Statistics Package for Social Sciences (SPSS; version 24). Negatively formulated items were quoted inversely. The students’ individual scores were analyzed according to the whole questionnaire as well as for each of the two notable components (perceived utility and enjoyment). A descriptive analysis (i.e., average and standard deviation) of the scores attained by the different groups of students was performed. Several MANOVAs were conducted to determine the differences in scores for the whole instrument depending on the grade level (i.e., 7th, 8th, and 9th grade), gender (male vs. female), socioeconomic status (i.e., upper, middle, and lower), and extracurricular sports practice (practice vs. no practice). MANOVA was used to analyze the differences in students’ global attitudes toward PE (dependent variable) based on their grade level (7th, 8th, and 9th grade), gender (female vs. male), socioeconomic status (upper, middle, and lower), and extracurricular sports practice (practice vs. no practice), which were treated as independent variables. If the MANOVA were statistically significant, discriminant analysis was conducted, specifically ANOVA, to determine differences between groups (Stevens, 2002). ANOVA was used to examine the influence of students’ grades on their attitudes toward PE.

## Results

### Validation

In the validation stage of the SAtPE, a subsample of 399 students was included (51.6% males and 48.4% females; mean age = 13.37; SD = 0.93). The variables presented univariate normality as the Sk and Ku values were lower than |1.15| e |1.23|, respectively (Table 1). Thus, there were no significant normality violations. According to Marôco (2014), |Sk| < 3 e|Ku| < 10 are considered acceptable.

**TABLE 1:** SAtPE’s descriptives per item.

**Factor**	**Item**	**Average**	**SD**	***Sk***	***Ku***	**λ**	**λ^2^**	**CR**	**AVE**
Perceived utility	4	3.96	1.32	–1.02	–0.28	0.53	0.28	0.81	0.54
	6	4.01	1.26	–1.14	0.18	0.56	0.32		
	7	4.03	0.99	–0.92	0.36	0.73	0.54		
	8	4.11	0.89	–0.43	0.73	0.80	0.64		
	10	3.70	1.31	−77	–0.47	0.57	0.32		
	13	3.88	1.05	–0.67	–0.05	0.70	0.49		
	14	3.43	1.39	–0.31	–1.22	0.48	0.23		
	16	3.75	1.30	–0.79	–0.46	0.55	0.30		
	17	4.10	0.95	–0.89	0.45	0.76	0.58		
	18	3.97	1.25	–1.01	–0.08	0.54	0.29		
Enjoyment	1	4.17	0.85	–0.65	–0.51	0.75	0.56	0.86	0.51
	2	3.96	1.22	–0.92	–0.23	0.49	0.24		
	3	4.09	0.93	–0.80	–0.02	0.73	0.54		
	5	3.99	1.23	–1.08	–0.15	0.57	0.33		
	9	4.09	0.91	–0.85	0.44	0.74	0.54		
	11	4.06	0.94	–0.68	–0.30	0.74	0.55		
	12	3.96	1.18	–0.93	–0.13	0.56	0.31		
	15	3.97	1.26	–0.96	–0.26	0.52	0.27		
	19	4.06	0.93	–0.89	0.64	0.75	0.57		
	20	4.14	0.87	–0.68	–0.15	0.77	0.59		

Concerning multivariate normality, Mardia’s coefficient was 39.53, which is lower than *p* (*p* + 2). The two-factor model tested through CFA presented acceptable fit indices [χ^2^ (342) = 1146.2; *p* < 0.001; χ^2^*/gl* = 3.3; GFI = 0.88; CFI = 0.91; NFI = 0.89; TLI = 0.89; RMSEA = 0.07; RMSR = 0.09]. To increase model adequacy, trajectories between the residuals of items 12 and 15 (i.e., factor enjoyment) as well as 14 and 18 (i.e., factor perceived utility) were included. Thus, the model adequacy was slightly higher than the original model (GFI = 0.86; AGFI = 0.82; RMSEA = 0.08; RMSR = 0.09). Therefore, we did not remove any item.

Table 1 shows data from standardized factorial weights, individual reliability (λ^2^), CR, and AVE. Most items have factorial weights higher than 0.50, which is considered acceptable (Hair et al., 2018). Only items 2 (“The games I learn in my physical education class make learning unpleasant for me”) and 14 (“The games I learn in my physical education class seem unimportant to me”) presented lower values of 0.49 and 0.48, respectively.

The two dimensions of “perceived utility” and “enjoyment” had high CR. Further, the AVE values were above the cutoff point of 0.5, which suggests good concurrent validity. Concerning the concurrent validity, the correlation between the square factors (0.522 = 0.027) was significantly lower than the AVE values for each factor. Thus, these factors are distinct.

The test-retest correlation was *r* = 0.869 for the entire questionnaire. Specifically, the coefficients for the perceived utility and enjoyment components were *r* = 0.828 and *r* = 0.826, respectively. These results showed that SAtPE had acceptable temporal stability.

### Descriptive Analysis

Students reported positive attitudes toward PE. The average score of most high school students concerning their attitudes toward PE was 79.86 (SD = 14.91). Male students obtained an average SAtPE score of 79.04 (SD = 15.63), while female students had an average score of 80.75 (SD = 14.04). Furthermore, the average scores obtained by students in the 7th, 8th, and 9th grades were 81.20 (SD = 13.81), 80.32 (SD = 13.57), and 76.54 (SD = 16.95), respectively (Table 2).

**TABLE 2:** Scores in the SAtPE according to the grade level and gender.

**Factor**	**7th grade (*n* = 151; 31.7%)**			**8th grade (*n* = 232; 48.7%)**			**9th grade (*n* = 93; 19.5%)**			**Total (*n* = 476; 100%)**		
	**Male**	**Female**	**Male + Female**	**Male**	**Female**	**Male + Female**	**Male**	**Female**	**Male + Female**	**Male**	**Female**	**Male + Female**
Enjoyment	41.89	41.46	41.70	39.29	42.10	40.66	38.84	39.70	39.25	40.06	41.43	40.71
Perceived usefulness	40.48	38.38	39.50	38.62	40.77	39.66	37.39	37.18	37.29	38.99	39.33	39.15
Total attitudes	82.37	79.81	81.20	77.91	82.87	80.32	76.22	76.89	76.54	79.04	80.75	79.86

Regarding socioeconomic status, low, middle, and high-class students had average scores of 78.05 (SD = 17.20), 81.03 (SD = 13.40), and 80.85 (SD = 12.51; Table 3), respectively.

**TABLE 3:** Scores in the SAtPE according to the socioeconomic status.

**Factor**	**Low (*n* = 183; 38.4%)**	**Middle (*n* = 227; 47.7%)**	**High (*n* = 66; 13.9%)**
Enjoyment	39.72 (8.53)	41.36 (7.15)	41.23 (6.27)
Perceived usefulness	38.33 (9.05)	39.67 (6.93)	39.62 (6.76)
Total	78.05 (17.19)	81.03 (13.40)	80.85 (12.51)

Of the sample, 62% participated in extracurricular sports activities. Regarding the grade level, 64.9, 67.2, and 44.1% of students from the 7th, 8th, and 9th grades, respectively, were involved in extracurricular sports activities. Furthermore, 65.5% of boys and 58.1% of girls practiced extracurricular sports activities. Concerning students’ participation in sports activities based on their socioeconomic status, findings showed that 60.6% of high class, 70.5% of middle class, and 51.9% of low-class students were involved in such activities. Students who practiced extracurricular sports activities had an average score of 82.75 (SD = 13.59) in the SAtPE, while those who did not practice averaged at 75.14 (SD = 15.76; Table 4).

**TABLE 4:** Scores in the SAtPE according to practice vs. non-practice of extracurricular sport activities.

**Factor**	**Do sports (*n* = 295; 62%)**	**Do not play sports (*n* = 181; 38%)**
Enjoyment	42.11 (7.02)	38.44 (7.90)
Perceived usefulness	40.65 (7.25)	36.71 (8.40)
Total	82.75 (13.59)	75.14 (15.76)

Regarding grades in PE (in Portuguese school settings, grades range from 1 to 5), students who attained a grade of 3 had an average score of 76.32 (SD = 14.14) in the SAtPE. Conversely, those who obtained a grade of 4 had an average of 81.91 (SD = 15.26), while those who attained 5 had an average of 84.50 (SD = 13.38; Table 5).

**TABLE 5:** Scores in the SAtPE according to grades in PE.

**Factor**	**Grade in PE – 3 (*n* = 184; 38.7%)**	**Grade in PE – 4 (*n* = 237; 49.8%)**	**Grade in PE – 5 (*n* = 44; 9.2%)**
Enjoyment	38.60 (7.32)	42.04 (7.64)	42.93 (7.17)
Perceived usefulness	37.72 (7.38)	39.87 (8.10)	41.57 (6.69)
Total	76.32 (14.14)	81.91 (15.26)	84.50 (13.38)

### Inferential Analysis

Generally, the groups of students mentioned above obtained higher scores in the enjoyment factor than in the perceived usefulness factor. Thus, Table 6 shows the results of MANOVA for students’ attitudes toward PE, considering the main effects of each of the four independent variables and their interaction effects.

**TABLE 6:** MANOVA for the students’ global attitude toward PE.

**Variables**	**MANOVA**				
	**Wilks’ λ**	***F***	***gl***	**Sig.**	**$\eta_{\text{p}}^{2}$**
**Grade level (GL)**	0.973	3.066	4	0.016	0.014
**Gender**	0.986	3.172	2	0.043*	0.014
**Socioeconomic status (ST)**	0.984	1.813	4	0.124*	0.008
**Sports practice (SP)**	0.967	7.559	2	0.001	0.033
GL × gender	0.992	0.833	4	0.504**	0.004
GL × ST	0.967	1.878	8	0.060	0.017
GL × SP	0.994	0.688	4	0.600	0.003
Gender × ST	0.994	0.669	4	0.613	0.003
Gender × SP	0.999	0.166	2	0.847	0.001
ST × SP	0.998	0.259	4	0.904	0.001
GL × gender × ST	0.980	1.135	8	0.336	0.01
GL × gender × SP	0.991	1.032	4	0.390	0.005
GL × ST × SP	0.973	1.539	8	0.140	0.014
Gender × ST × SP	0.983	1.898	4	0.109	0.009
GL × gender × ST × SP	0.983	1.286	6	0.261	0.009

*p < 0.05, **p < 0.01.

Multivariate analysis showed statistically significant univariate effects of grade level [Wilk’s lambda = 0.973, *F*_(4, 880)_ = 3.066, *p* < 0.05, $\eta_{\text{p}}^{2}=0.014$], gender [Wilk’s Lambda = 0.986, *F*_(2, 440)_ = 3.172, *p* < 0.05, $\eta_{\text{p}}^{2}=0.014$], and extracurricular sports activities [Wilk’s Lambda = 0.967, *F*_(2, 440)_ = 7.559, *p* < 0.01, $\eta_{\text{p}}^{2}=0.033$] on students’ global attitude toward PE. In contrast, students’ socioeconomic status did not influence their attitudes toward PE. There were no significant interaction effects between the four independent variables (grade level, gender, socioeconomic status, and extracurricular sports participation).

Concerning the grade level, discriminant analysis showed differences only in the perceived utility factor of attitudes [*F*_(__2__)_ = 3.055, *p* < 0.05, $\eta_{\text{p}}^{2}=0.013$]. ANOVA indicated that students from the eighth-grade had significantly higher scores on enjoyment and perceived utility factors than their ninth-grade counterparts. Concerning gender, univariate analysis highlighted statistically significant differences between male and female students regarding the affective factor of attitudes toward PE [*F*_(__1__)_ = 3.863, *p* ≤ 0.05, $\eta_{\text{p}}^{2}=0.08$], with the latter obtaining higher values. Regarding extracurricular sports practice, univariate analysis showed that statistically significant differences were associated with enjoyment [*F*_(__1__)_ = 27.412, *p* < 0.01, $\eta_{\text{p}}^{2}=0.055$] and perceived utility [*F*_(__1__)_ = 30.36, *p* < 0.01, $\eta_{\text{p}}^{2}=0.060$] factors of the SAtPE.

Finally, we examined the relationship between students’ school grades and their attitudes toward PE. Through ANOVA, the data obtained showed statistically significant differences in attitudes toward PE [F_(__3__)_ = 6.673, *p* < 0.01, $\eta_{\text{p}}^{2}=0.041$] and in its two factors, enjoyment [*F*_(__3__)_ = 8.924, *p* < 0.01, $\eta_{\text{p}}^{2}=0.054$] and perceived utility [*F*_(__3__)_ = 4.234, *p* < 0.01, $\eta_{\text{p}}^{2}=0.026$], depending on the students’ grades. Scheffe’s *post hoc* test showed that these differences were evident between students who had grades of 4 and 5 in PE.

## Discussion

This study examined Portuguese high school students’ attitudes toward PE. The SAtPE was validated to achieve this objective. Our findings showed that the instrument presented acceptable psychometric properties concerning validity and reliability and may be used to assess Portuguese high school students’ attitudes toward PE, which provides new opportunities for researchers to explore this line of inquiry. However, it is essential to continue testing the instrument either by using different samples or examining whether the four-factor structure imparts a more appropriate adjustment.

Our findings showed that students generally had a moderately positive attitude toward PE, which confirms hypothesis (1). Specifically, our study suggests that students enjoy PE classes and consider it an important discipline. Thus, Portuguese students’ scores in the SAtPE were slightly higher than those reported in previous studies conducted in the United States (Subramaniam and Silverman, 2007; Scrabis-Fletcher and Silverman, 2017; Marttinen et al., 2018), Europe (Lazarević et al., 2015), and Asia (Hu et al., 2014), despite having a similar sample. Research has shown that positive attitudes toward PE are associated with the nature of this discipline. Moreover, several researchers (Delfosse et al., 1997; Piéron, 2005) have stated that PE is a practical discipline wherein social interaction, fun, freedom, and movement-play are crucial, as opposed to other disciplines in the curriculum.

The findings confirmed hypothesis (2), which was centered around the association between grade level and attitudes toward PE. Thus, we found that students’ positive attitudes toward PE decreased throughout high school, especially in the 9th grade. This decrease may derive from the cognitive component that is associated with the perceived usefulness of PE, which in turn largely depends on the curriculum and the PE teacher. Our results are corroborated by previous studies (Subramaniam and Silverman, 2000, 2007; Hu et al., 2014; Lazarević et al., 2015; Scrabis-Fletcher et al., 2016; Mercier et al., 2017; Evangelou and Digelidis, 2018; Marttinen et al., 2018) that show that students’ attitudes toward PE become less positive as they age. However, in these studies, this decline in positive attitudes toward PE is predominantly related to the affective component, which was not the case in our study.

It is of utmost importance for researchers, policymakers, and, particularly, PE teachers to understand the reasons for the decrease in positive attitudes toward PE. This is paramount because students’ attitudes toward PE impact their participation in extracurricular activities and sports, and the creation of a foundation for active lifestyles (Solmon and Lee, 1996; Kohl and Hobbs, 1998; Hagger et al., 2003; McKenzie, 2003; Subramaniam and Silverman, 2007). One of the reasons for the decrease in positive attitudes toward PE is related to the fact that, in some cases, the curriculum lacks meaningful content that challenges students to increase their motor proficiency within an intrinsically motivated environment (Carlson, 1995; Subramaniam and Silverman, 2007). In Portugal, PE teachers are pressured to teach an extensive range of lessons (Santos et al., 2020) throughout grade levels, which may influence the way they use curriculum ownership and engage with students (Carlson, 1995; Subramaniam and Silverman, 2007). It is necessary to be informed regarding curricular reforms, consider students’ attitudes toward PE, and use enjoyment and perceived utility as guiding variables for further development (Subramaniam and Silverman, 2000, 2002, 2007; Rikard and Banville, 2006; Montalvo and Silverman, 2008; Silverman, 2011; Phillips and Silverman, 2015; Mercier et al., 2017; Scrabis-Fletcher and Silverman, 2017; Subramaniam and Mercier, 2017; Marttinen et al., 2018; Orlić et al., 2018). Curriculum ownership has been associated with an intrinsically motivated environment (Farias et al., 2020). Specifically, several factors have been considered crucial in fostering a positive climate in PE: (a) a task-oriented climate, (b) the integration of learner-centered models, such as the sports education model and a variety of teaching styles, and (c) the promotion of enjoyment, competency, and positive social relationships (Siedentop, 1994, 2004; Subramaniam and Silverman, 2002, 2007; Digelidis et al., 2003; Silverman, 2011; Mercier et al., 2017; Scrabis-Fletcher and Silverman, 2017; Constantinides and Silverman, 2018; Orlić et al., 2018).

Previous research has also indicated that as students get older, they consider the PE curriculum to be less useful (Constantinides and Silverman, 2018). In Portugal, students in the 9th grade must take exams in a range of disciplines. The pressure to perform and attain high scores in a vast array of disciplines may also influence how students experience PE, which may ultimately result in a lack of enjoyment. It is possible that as students get older, they undertake more demanding performance objectives and go through a set of social forces that value grades in other areas, which may result in reducing the importance of PE.

Hypotheses (3) and (4) were not confirmed. Our findings also showed that female students had more favorable attitudes toward PE than their male counterparts. However, these findings do not support previous studies conducted in the context of high school wherein male students reported having more positive attitudes toward PE (Lazarević et al., 2015; Mercier et al., 2017), or with no statistically significant differences between genders (Subramaniam and Silverman, 2000, 2007; Hu et al., 2014; Scrabis-Fletcher et al., 2016; Marttinen et al., 2018; Orlić et al., 2018). In this study, female students reported significantly higher scores in the affective component, which suggests more enjoyment in PE.

Our findings might have been derived from the way the curriculum in Portugal is currently framed. The PE curriculum involves a range of physical and sports activities, such as dance and badminton. Previous research has shown that female students have more positive attitudes toward activities that value esthetic dimensions (e.g., dance, gymnastics), which are an important part of the PE curriculum, while male students present a more favorable attitude in activities that involve taking on challenges and risks, such as football (Greenwood and Stillwell, 2001; Zeng et al., 2011). Notably, the gender differences in attitudes toward PE may also be derived from cultural and societal value systems. From the perspective of several researchers (Tannehill et al., 1994; Piéron et al., 1997), students’ attitudes toward PE are influenced by self-image, family, and social media, which create expectations for motor skill development and performance in various physical activities and sports.

As for the mediating role of students’ socioeconomic status, our findings suggest that this variable did not influence attitudes toward PE, as indicated by previous research, which in turn did not confirm hypothesis (4) (Zeng et al., 2011). Our findings suggest that PE contributes to minimizing differences in attitudes among students from different socioeconomic statuses, which is an important variable for participation in extracurricular sports activities (Johnston et al., 2007).

Students who engaged in extracurricular sports activities had a more positive attitude toward PE than those who did not, as supported by previous research (Lazarević et al., 2015; Peralta et al., 2015; Lima et al., 2018; Orlić et al., 2018). This finding confirmed hypothesis (5). Although we did not identify a cause-effect relationship between the two variables, our findings suggest that students have more positive attitudes toward PE when they engage in extracurricular sports activities, which is aligned with the existing literature in this field (Phillips and Silverman, 2015). Furthermore, it was found that students’ attitudes toward PE were influenced by their grades, as suggested by Li et al. (2014) and Orlić et al. (2018). This confirmed hypothesis (6). In other words, students with better grades had a more positive attitude toward PE. Grades have been acknowledged as a mediating variable of attitudes toward PE (Subramaniam and Silverman, 2007). It is noteworthy that there were no significant interaction effects in differentiating students’ attitudes toward PE, which requires further exploration.

## Conclusion

Positive attitudes toward PE may serve as motivation for PE teachers to achieve the final objective of PE—to foster participation in extracurricular sports and encourage engagement in physical activities throughout life. This study adds to the literature by shedding light on the attitudes of Portuguese high school students. However, this study has several limitations. First, a non-probabilistic sample was used in this study. Second, the sample was not representative of the Portuguese high school context, since only students from the north and center of the country were included. Finally, the model adequacy was only considered acceptable, which may suggest the need for further validation efforts.

As the field progresses, more descriptive and longitudinal studies must be conducted in European education systems to understand how students experience PE. Within this line of inquiry, it is essential to use validated tools and theory-based approaches to deductively characterize students’ attitudes toward PE. Describing the variables that are responsible for the increase or decrease in attitudes toward PE could also provide valuable insight for stakeholders and educational institutions. Further studies should also examine the effects of continuing professional development programs on students’ attitudes toward PE, which have implications on how to train PE teachers. More Portuguese researchers should follow this line of study, which is paramount for generating sound and evidence-based policy and practice in the coming years.

## Data Availability Statement

The raw data supporting the conclusions of this article will be made available by the authors, without undue reservation.

## Ethics Statement

The studies involving human participants were reviewed and approved by the Portuguese Ministry of Education (Office of Statistics and Education Planning). Written informed consent to participate in this study was provided by the participants’ legal guardian/next of kin.

## Author Contributions

PP: defining purpose, rationale, data collection and analysis, and writing. FS: data collection, revisions, rationale, and writing. DM: revisions and writing. All authors contributed to the article and approved the submitted version.

## Conflict of Interest

The authors declare that the research was conducted in the absence of any commercial or financial relationships that could be construed as a potential conflict of interest.

## Appendix

**Table:**

**Item**	**Factor**
1. The games I learn in physical education make my physical education class interesting for me. Os jogos que aprendo na aula de educação física fazem com que a aula de educação física seja interessante para mim.	*Factor 1: Enjoyment*
2. The games I learn in my physical education class make learning unpleasant for me. Os jogos que aprendo na aula de educação física tornam a aprendizagem algo desagradável para mim.	
3. The games I learn in my physical education class get me excited about physical education. Os jogos que aprendo na aula de educação física fazem com que fique entusiasmado em relação à educação física.	
5. I feel the games I learn in physical education make my physical education class boring for me. Eu sinto que os jogos que aprendo na educação física fazem com que a aula de educação física seja aborrecida.	
9. My physical education teacher makes my physical education class interesting for me. O meu professor de educação física faz com a aula de educação física seja interessante para mim.	
11. I feel my physical education teacher makes learning in my physical education class fun for me. Eu sinto que o meu professor de educação física faz com que aprender na aula de educação física seja divertido para mim.	
12. I feel my physical education teacher makes my physical education class boring for me. Eu sinto que o meu professor de educação física faz com que a aula de educação física seja aborrecida para mim.	
15. My physical education teacher makes learning in my physical education class unpleasant for me. Eu sinto que o meu professor de educação física faz com que aprender na aula de educação física seja desagradável para mim.	
19. My physical education teacher gets me excited about physical education. O meu professor de educação física faz com que fique entusiasmado com a educação física.	
20. I feel the games I learn in my physical education class make learning fun for me. Eu sinto que os jogos que aprendo na aula de educação física fazem com que aprender seja divertido para mim.	
4. My physical education teacher makes my physical education class seem unimportant to me. O meu professor de educação física faz com que as aulas de educação física não sejam importantes para mim.	*Factor 2: Usefulness*
6. I feel the games I learn in my physical education class are useless to me. Eu sinto que os jogos que aprendo na aula de educação física são inúteis para mim.	
7. The games I learn in my physical education class seem important to me. Eu sinto que os jogos que aprendo na aula de educação física são importantes para mim	
8. My physical education teacher makes my physical education class seem important to me. O meu professor de educação física faz com que as aulas de educação física sejam importantes para mim.	
10. The games I learn in my physical education class are useful to me. Eu sinto que os jogos que aprendo na aula de educação física são úteis para mim.	
13. I feel the games I learn in my physical education class are valuable to me. Eu sinto que os jogos que aprendo na aula de educação física são essenciais para mim.	
14. The games I learn in my physical education class seem unimportant to me. Eu sinto que os jogos que aprendo na aula de educação física não são importantes para mim.	
16. My physical education teacher makes my physical education class useful for me. Eu sinto que o meu professor de educação física faz com que aprender na aula de educação física seja útil para mim.	
17. I feel my physical education teacher makes learning in my physical education class valuable for me. Eu sinto que o meu professor de educação física faz com que aprender na aula de educação física seja essencial para mim.	
18. I feel my physical education teacher makes learning in my physical education class useless for me. Eu sinto que o meu professor de educação física faz com que aprender na aula de educação física seja inútil para mim.	
