# Corrigendum: Examining Portuguese High School Students' Attitudes Toward Physical Education

**DOI:** 10.3389/fpsyg.2021.662081

**Published:** 2021-02-26

**Authors:** Paulo Pereira, Fernando Santos, Daniel A. Marinho

**Affiliations:** ^1^School of Higher Education, Polytechnic Institute of Porto, Porto, Portugal; ^2^School of Higher Education, Polytechnic Institute of Viana Do Castelo, Viana Do Castelo, Portugal; ^3^Department of Sport Sciences, University of Beira Interior, Covilhã, Portugal; ^4^Research Centre in Sports, Health and Human Development, CIDESD, Covilhã, Portugal

**Keywords:** school, pedagogy, curriculum, extracurricular sports, physical activity

In the original article, there were errors in the affiliations as published.

There were errors regarding the numbering of the affiliations, and two affiliations were previously omitted. The corrected affiliations and numbering are shown below:

Paulo Pereira^1^^*^, Fernando Santos^**1,2**^**, Daniel A. Marinho**^**3,4**^

^**1**^ ***School of Higher Education, Polytechnic Institute of Porto, Porto, Portugal***

^**2**^ ***School of Higher Education, Polytechnic Institute of Viana do Castelo, Viana do Castelo*,** ***Portugal***

^**3**^ ***Department of Sport Sciences, University of Beira Interior, Covilh*ã*, Portugal***

^**4**^ ***Research Centre in Sports, Health and Human Development, CIDESD, Covilh*ã,** ***Portugal***

The authors apologize for these errors and state that they do not change the scientific conclusions of the article in any way. The original article has been updated.

